# miRNA profiling, detection of BRAF V600E mutation and RET-PTC1 translocation in patients from Novosibirsk oblast (Russia) with different types of thyroid tumors

**DOI:** 10.1186/s12885-016-2240-2

**Published:** 2016-03-09

**Authors:** Sergei E. Titov, Mikhail K. Ivanov, Elena V. Karpinskaya, Elena V. Tsivlikova, Sergei P. Shevchenko, Yulia A. Veryaskina, Larisa G. Akhmerova, Tatiana L. Poloz, Olesya A. Klimova, Lyudmila F. Gulyaeva, Igor F. Zhimulev, Nikolay N. Kolesnikov

**Affiliations:** 1 Institute of Molecular and Cellular Biology, SB RAS, Novosibirsk, Russia; 2 JSC “Vector-Best”, Koltsovo, Russia; 3 Novosibirsk Municipal Budgetary Healthcare Institution “Municipal Clinical Hospital #1”, Novosibirsk, Russia; 4 Non-governmental Healthcare Institution «Railroad Clinical Hospital on the Station Novosibirsk-Glavny”, JSC Russian Railways, Novosibirsk, Russia; 5 Institute of Molecular Biology and Biophysics, SB RAMS, Novosibirsk, Russia

**Keywords:** Thyroid cancer, microRNA, BRAF, RET-PTC1, Real-time PCR

## Abstract

**Background:**

The postoperative typing of thyroid lesions, which is instrumental in adequate patient treatment, is currently based on histologic examination. However, it depends on pathologist’s qualification and can be difficult in some cases. Numerous studies have shown that molecular markers such as microRNAs and somatic mutations may be useful to assist in these cases, but no consensus exists on the set of markers that is optimal for that purpose. The aim of the study was to discriminate between different thyroid neoplasms by RT-PCR, using a limited set of microRNAs selected from literature.

**Methods:**

By RT-PCR we evaluated the relative levels of 15 microRNAs (miR-221, −222, −146b, −181b, −21, −187, −199b, −144, −192, −200a, −200b, −205, −141, −31, −375) and the presence of BRAF(V600E) mutation and RET-PTC1 translocation in surgically resected lesions from 208 patients from Novosibirsk oblast (Russia) with different types of thyroid neoplasms. Expression of each microRNA was normalized to adjacent non-tumor tissue. Three pieces of lesion tissue from each patient (39 goiters, 41 follicular adenomas, 16 follicular thyroid cancers, 108 papillary thyroid cancers, 4 medullary thyroid cancers) were analyzed independently to take into account method variation.

**Results:**

The diagnostic classifier based on profiling of 13 microRNAs was proposed, with total estimated accuracy varying from 82.7 to 99 % for different nodule types. Relative expression of six microRNAs (miR-146b, −21, −221, −222, 375, −199b) appeared significantly different in BRAF(V600E)-positive samples (all classified as papillary thyroid carcinomas) compared to BRAF(V600E)-negative papillary carcinoma samples.

**Conclusions:**

The results confirm practical feasibility of using molecular markers for typing of thyroid neoplasms and clarification of controversial cases.

**Electronic supplementary material:**

The online version of this article (doi:10.1186/s12885-016-2240-2) contains supplementary material, which is available to authorized users.

## Background

Nodular thyroid lesions are the most frequent endocrine pathology. Thyroid nodules are diagnosed in over 5 % of the adult population and can be subdivided into benign adenomas or malignant lesions (carcinomas). Carcinomas, requiring mandatory surgery, only make up about 5 % of all neoplasms.

Carcinomas are derived from two types of hormone-producing cells; follicular cells and parafollicular C-cells. More than 95 % of the thyroid carcinomas originate from follicular cells and can be grouped into three main categories: papillary thyroid carcinoma (PTC), follicular thyroid carcinoma (FTC) and anaplastic thyroid carcinoma (ATC). Medullary thyroid carcinomas (MTC) are derived from the parafollicular C-cells, they account for a minority (3 %) of thyroid carcinomas [1, 2].

Post-operative histopathology examination of resected thyroid samples is instrumental to making correct definitive diagnosis. This analysis is never 100 % accurate and heavily relies on the pathologist’s qualification and experience. The problem is exacerbated by the fact that accurate histopathological examination requires professional performance of biopsy sampling and a large number of well-prepared histological micropreparations, which is not always achievable in routine practice. Thus, there is a pressing need for improving post-operative subtyping of thyroid nodules. Clearly then, identification of specific molecular markers should help to increase the robustness and objectivity of typing. The most recognized markers in current use are somatic mutations (BRAF V600E, mutations of isoforms RAS) and translocations (RET-PTC1, PAX8-PPAR γ). However, not always somatic changes enabling the accurate classification of neoplasms can be identified. MicroRNAs (miRNAs) can also serve as such markers, since recent studies showed that expression of many of these is always subject to profound changes in various types of thyroid neoplasms (Table 1).

**Table 1:** Deregulation of miRNA expression in different types of thyroid tumors according to some literature data

Cancer type	miRNA	Expression	Detection method	Reference
Papillary thyroid carcinoma (PTC)	**187, 221, 222, 146b,** 155, 122a, **31, 205,** 224	Up-regulated	RT-qPCR	[3]
	**146b, 181b, 21, 221, 222**	Up-regulated	RT-qPCR	[4]
	**221, 222**	Up-regulated	RT-qPCR	[5]
	**146, 221, 222**	Up-regulated	microarray, RT-qPCR	[6]
	**221, 222, 181b**	Up-regulated	microarray, RT-qPCR	[7]
	**146b, 221, 222**	Up-regulated	RT-qPCR	[8]
	**221, 222, 21, 31, 181b**, 223, 224	Up-regulated	microarray, RT-qPCR	[9]
	218, 300, 292, 345, 30c	Down-regulated	microarray, RT-qPCR	[9]
Follicular variant of papillary carcinoma (FVPTC)/PTC	146b-3p, **146-5p**, **221, 222 375**, 551b, 181-2-3p, 99b-3p	Up-regulated	microarray, RT-qPCR	[10]
Medullary thyroid cancer (MTC)	323, 370, 129, 137, 10a, 124a, 224, 127, 9, 154	Up-regulated	RT-qPCR	[3]
	183, **375**	Up-regulated	RT-qPCR	[11]
Follicular thyroid carcinoma (FTC)	**187,** 224**, 221**, 339, 183, **222**	Up-regulated	RT-qPCR	[3]
	**199b-5p, 144-5p**, 144-3p, 199b-3p	Down-regulated	microarray, RT-qPCR	[12]
	**192**, 197, 328, 346	Up-regulated	microarray, RT-qPCR	[13]
Thyroid follicular adenoma (FA)	**31,** 339, 183, **221**, 224, **205**, 210, 190	Up-regulated	RT-qPCR	[3]
	**199b-5p, 144-5p**, 663, 199b-3p	Down-regulated	microarray, RT-qPCR	[12]

miRNAs shown in boldface type were used in our work

Three things should be kept in mind: first, even though it was shown that concentration changes of several miRNAs may serve as informative indicators of thyroid nodule malignancy itself, these values are not sufficiently informative for accurate typing of malignant neoplasms (for instance, see [14, 15]); second, lists of miRNAs demonstrating significant expression changes in specific thyroid neoplasms show limited overlap in different reports. This may partially be attributable to both differing efficiencies of detection methods used (as in [16]) and to biases and errors intrinsic to histological and immunohistochemical analysis serving as a reference. Third, a recent study [17] revealed the presence of several discrete subclasses of PTCs, some of these subclasses, statistically less often represented in the total sample, were characterized by expression profiles significantly different from the others.

The purpose of this work is to develop a classifier based on the PCR quantification of a limited number of microRNAs in the surgically excised thyroid tissue. Such an analysis used in addition to histological examination is intended for improving the reliability of typing thyroid neoplasms, including clarification of ambiguous results of histological analysis. Therefore, we planned to pay special attention to controversial cases, where the result of the analysis with the help of the developed classifier was at variance with the result of the histological report.

For this purpose, we planned to assess the difference in the content of several pre-selected miRNAs in the samples from patients with different types of thyroid tumors identified histopathologically; and based on the analysis of the results to develop miRNA classifier for molecular subtyping of thyroid neoplasms.

According to the literature data, increased expression of miR-146b, −221, −222, −181b, and −21 is characteristic of PTC. These, as well as miR-187, were selected as candidates in our work aimed at increasing the accuracy of differential diagnosis of papillary cancer. Fewer data are available for follicular thyroid carcinoma (FTC) and follicular adenoma (FA), and so the major goal here is to discriminate reliably between FA and the rest of the benign nodules. Based on the information presented in Rossing et al. [12], we selected miR-199b-5p and 144-5p so as to enable differential diagnosis of follicular tumors. Next, miR-192 [13] was chosen as a marker that would help discriminate between FTC and FA. Several additional miRNAs were also tested, based on the pronounced histological difference between these tumor types, namely capsular and/or vascular invasion, characteristic of follicular thyroid carcinoma [18]. Follicular subtype is also relatively more prone to metastasis. This property largely results from the invasiveness of cancer cells, which in turn relies on the process of epithelial-mesenchymal transition (EMT). Thus, the above-mentioned additional miRNAs included miR-200a/b, −205, −141-3p and −31, as the former two were reported to control EMT [19, 20] whereas miR-200 is also known to inhibit angiogenesis [21]. miR-141-3p was chosen for it belongs to the same family as miR-200, and miR-31 was demonstrated to negatively regulate cell invasiveness [22]. Finally, consistent with the recent findings, miR-375 was selected as a candidate marker for differential diagnosis of medullary thyroid carcinoma (MTC) [11]. Thus, our final set of miRNAs included miR-221-3p, −222-3p, −146b-5p, −181b-5p, −21-5p, −187-3p, −199b-5p, −144-5p, −192-5p, −200a-3p, −200b-3p, −205-5p, −141-3p, −31-5p, −375.

As additional markers, we intended to use the detection of point mutation in BRAF (V600E) and RET-PTC1 translocation, which most frequently occur in patients with PTC. In the case of contradictory results (a fundamental difference in the histological report and identification of a neoplasm made by the miRNA classifier) detection of one of these markers in the sample may confirm the malignancy of the neoplasm.

## Methods

### Clinical samples

This study was approved by the ethics committee of the Institute of Molecular Biology and Biophysics, Siberian Branch of the Russian Academy of Medical Sciences. Surgical material was obtained in compliance with the legislation of the Russian Federation, and written informed consent was provided by all the patients. 208 tissue samples from thyroid nodules were surgically resected from patients undergoing thyroidectomy (28 men and 180 women, median age 54 and 56 years, respectively). The samples, collected between 2011 and 2013, represent a workflow of a house surgeon during about 2.5 years. Samples had not been pre-selected intentionally, so the proportion of lesion types reflects the real distribution of thyroidectomy cases in the setting of study. Nevertheless, due to various reasons, mostly technical, 12 patients were excluded from analysis, including one case of anaplastic thyroid carcinoma and one case of poorly differentiated squamous cell carcinoma. Adjacent non-tumor thyroid tissue served as a control. Special care was taken to ensure that no adjacent normal tissue was present in the tumor sample, and vice versa. Sample collection and histology analysis were controlled by a qualified oncologist (Oncology department VI, Novosibirsk Municipal Clinical Hospital #1). Upon resection, tissue samples were immediately placed in EverFresh RNA solution (SILEX, Russia) and stored at +4-8 °C for up to a week until processed. Demographic and clinical characteristics of the samples are shown in [Additional file 1].

### Histopathology analysis

Tissue samples were processed according to the standard protocol, i.e., tumor pieces and regional lymph nodes were fixed in 10 % neutral buffered formalin, dehydrated in a graded series of alcohols, cleared in xylol and embedded in paraffin. 5 μm thick paraffin sections were stained with hematoxylin and eosin. This was followed by light microscopy imaging (5 slides per sample on average). Analysis of all samples was carried out by staff histologists of the “Municipal Clinical Hospital No. 1”, we used the diagnoses given to the patients. Some samples were independently analyzed at the Railroad Clinical Hospital (JSC Russian Railways, Novosibirsk) by an independent expert with work experience of more than 20 years.

### RNA isolation

500 μL of guanidine lysis buffer (4 M guanidine isothiocyanate, 25 mM sodium citrate, 0.3 % sarkosyl, 3 % DTT aliquoted in oxygen-free atmosphere, supplied by Vector-Best, Russia) was added to 50 mg of tissue. The sample was vigorously mixed and left in a thermal shaker for 15 min at 65 °C. Next, the tube was centrifuged at 10000 g for 2 min, the supernatant was transferred into new vials, followed by addition of an equal volume of isopropanol. Reaction was thoroughly mixed and left at room temperature for 5 min. After centrifugation for 10 min at 12000 g, the supernatant was discarded, and the pellet was washed with 500 μL 70 % ethanol and 300 μL acetone. Finally, the RNA was dissolved in 200 μL of deionized water. If not analyzed immediately, RNA preparations were stored at −20 °C until further use.

### miRNA detection

To quantify miRNA, we followed the protocol published by Chen and co-authors in 2005 as it allows highly sensitive and specific identification of mature miRNAs. This protocol includes reverse transcription of mature miRNA using long stem-loop primer, which is followed by detection of cDNA via RT-qPCR [23]. Reverse transcription reactions were set individually for each miRNA to be quantified. The obtained cDNA was used for further PCR analysis immediately.

### Synthetic analogs of miRNAs

Synthetic analogs of miRNAs were ordered from Biosan (Russia) and stored frozen in TE at −20 °C until needed. When used as controls, miRNA analogs were dissolved in deionized water and added directly into RT reaction mix, omitting the purification/isolation steps.

### Oligonucleotide primers and probes

All oligonucleotides, including dual-labeled probes, were produced by Vector-Best (Russia). Oligonucleotide sequences were designed using online software tool PrimerQuest (Integrated DNA Technologies, USA). Several sets of primer and probe combinations were designed for each miRNA, and those showing high reverse transcription and PCR efficiencies were used for all downstream analyses. Efficiency of reverse transcription was assessed using quantification cycle (Cq) values obtained on synthetic miRNA analogs with known concentrations. Amplification efficiency (E) for each primers/probe combination was calculated by plotting a calibration curve over a series of RNA dilutions, with RNA isolated from clinical samples showing high levels of the miRNA of interest. E values ranged from 83.5 to 98.5 % for different miRNA amplifications. Sequences of primers and the probe are listed in Additional file 2. Sets of oligos for miR-146b, −181b −221 detection were validated by commercially available reagents TaqMan MicroRNA Assays (Applied Biosystems, USA). Within the linear range, the difference between Cq of our systems and Applied Biosystems not exceed 2. The difference in the efficiency of the reaction did not exceed 3.5 %.

### Reverse transcription

Total volume of each reaction was 30 μL. Reaction mix contained 3 μL of RNA preparation, 21.6 % trehalose, 1x RT buffer (Vector-Best, Russia), 0.4 mM of each dNTP, 1 % BSA, 100U M-MLV reverse transcriptase (Vector-Best, Russia), 0.2 μM of appropriate RT primer. Reaction was incubated for 15 min at 16 °C and 15 min at 42 °C, which was followed by heat inactivation for 2 min at 95 °C. 3 μL of RT mix was used per one RT-qPCR reaction.

### Real-time PCR

Real-time PCR was performed using CFX96 thermal cycler (Bio-Rad Laboratories, USA). All reactions were set up manually. Total volume of each reaction was 30 μL and encompassed 3 μL of cDNA, 1x PCR buffer (Vector-Best, Russia), 0.4 mM of each dNTP (Biosan, Russia), 1 % BSA, 1U Taq polymerase (Vector-Best, Russia) pre-mixed with active center-specific monoclonal antibody (Clontech, USA), 0.5 units of uracil-DNA glycosylase (Vector-Best), 0.5 μM of each primer and 0.25 μM of dual-labeled probe. PCR cycling conditions were as follows: 2 min incubation at 50 °C, pre-denaturation step at 94 °C – 2 min, followed by 50 cycles of denaturation (94 °C for 10 s), annealing and elongation (60 °С for 20 s).

U6 snRNA served as an internal normalization control because it is most commonly used for this purpose in studies on the expression of miRNA [24]. Fold change in expression of each miRNA in the tumor vs normal tissue was calculated using the following formula which takes amplification efficiencies of each PCR into account [25]:

$$ \frac{C_{tumor}}{C_{norm}}=\frac{\frac{{\left(1+{E}_{U6}\right)}^{Cq,U6(tumor)}}{{\left(1+{E}_{miR}\right)}^{Cq,miR(tumor)}}}{\frac{{\left(1+{E}_{U6}\right)}^{Cq,U6(norm)}}{{\left(1+{E}_{miR}\right)}^{Cq,miR(norm)}}} $$

E stands for amplification efficiency of miRNA or U6 internal control, and Cq - quantification cycle.

Reactions with Cq above 37 were considered as negatives. All experimental runs included NTCs (non-template controls) for each miRNA analyzed. NTC’s were run in triplicates. In our hands and with the equipment used, the Cq values for NTC’s always exceeded 37.

For each patient, miRNAs were profiled in 3 different samples from thyroid tumor tissue and in 3 different samples of matching adjacent non-tumor tissue; average values were taken into analysis.

### Detection of BRAF(V600E)

Detection of BRAF(V600E) mutation was performed using allele-specific PCR with dual-labeled probe. Sequences of primers and the probe are listed in Additional file 2. PCR cycling conditions were as follows: pre-denaturation step 95 °С – 2 min, followed by 50 cycles of denaturation (94 °С, 10 s), annealing and elongation (60 °С, 15 s). Sensitivity of mutant allele detection, as assessed using samples with known wild-type and mutant sequences, was 0.75 % (data not shown). In cases where a mutation was detected, but no histology report stated “papillary carcinoma,” the presence of the mutation was confirmed by massive parallel sequencing on the platform 454 Junior (Roche).

### Sequencing

#### Design of libraries of amplicons and sequences of primers for analysis with Roche GS Junior

The amplification of the BRAF gene was carried out in two rounds. In the first round fusion primers were used, consisting of sequences flanking BRAF V600E mutation (GATCCAGACAACTGTTCAAAC and ATCTCATTTTCCTATCAGAGCAA), and attached to their 5′-ends adaptor sequences “U13” (GCGGTCCAAAAGGGTCAGT) and “U9” (TTAATATTGCCACGGGCCTA). In the second PCR round fusion primers were used, consisting of auxiliary sequences of the Junior platform, and “U13” and “U9”, located at their 3′ends.

#### Sequencing

The sequencing was performed on the GS Junior instrument using Titanium kits and following the standard 200 nucleotide flows protocol according to the manufacturer’s recommendations.

#### Data analysis

The initial data analysis was carried out using software of the manufacturer (GS Run Processor, Roche), according to the preconfigured set of filters “Amplicons”, and then using application software Amplicon Variant Analyzer v. 3.0 (Roche).

### Detection of RET-PTC1 translocation

Detection was performed using Real-time PCR combined with reverse transcription reaction in a single tube. Total volume of each reaction was 30 μL. Reaction mix contained 3 μL of RNA preparation, 16.7 % trehalose, 1x RT-PCR buffer (Vector-Best, Russia), 0.4 mM of each dNTP, 1 % BSA, 100U M-MLV reverse transcriptase (Vector-Best, Russia), 1U Taq polymerase (Vector-Best, Russia) pre-mixed with active center-specific monoclonal antibody (Clontech, USA), 0.5 μM of each primer and 0.25 μM of dual-labeled probe. RT-PCR protocol: incubation at 45 °С - 30 min., heating at 95 °С - 2 min., 50 cycles of denaturation at 94 °С - 10 s, annealing and elongation: 60 °С - 20 s. Sequences of primers and the probe are listed in Additional file 2.

## Results

### Measurements of miRNA levels in thyroid neoplasm vs adjacent non-tumor tissue

Histological report classified the 208 surgical samples as follows: goiter - 39 samples, FA – 41 samples, FTC – 16 samples, PTC – 108 samples (out of them 16 were FVPTC and the rest were a classical variant of PTC), MTC – 4 samples. The distribution of different types of tumors over the sampling with a clear predominance of papillary cancer reflected a real flow of samples sent to the pathomorphology examination.

Median fold changes observed for each of the miRNAs tested in tumor vs matching non-tumor tissue control from the same patient are summarized in Table 2 (see also Additional file 3, where data for individual patients are provided). These data clearly indicate that relative levels of miRNAs in neoplasms are indeed subject to extensive changes, and magnitude of those changes may be correlated with the specific neoplasm subtype. For instance, increased relative expression of miR-221, −222, −146b, −21, −181b and −187 was reported elsewhere for classic PTC, and this was indeed observed in our experiments. Pronounced fold changes in expression of miR-221, −222 and -146b were found in most PTCs, whereas miR-21 and -181b showed increased expression in about one third of cases. The increased level of miR-21 was observed in 38 out of 92 (41 %) samples classified as PTC, and in 3 out of 16 (19 %) samples classified as FVPTC. The increase of the miR-181b level was observed in 27 out of 92 (29 %) samples classified as PTC, and in 4 out of 16 (25 %) samples classified as FVPTC. miR-375 is known to be heavily overexpressed in MTC and also in PTС [10]. Our data are in line with these observations, we found that miR-375 displayed increased expression in about half of PTC samples. miR-199b and −144 were supposed to serve as markers of follicular neoplasms, which we do not confirm. Nor do our results confirm miR-192 as a marker of FTC, as this miRNA showed little expression changes across tumor types. Finally, in order to discriminate between follicular thyroid adenomas and carcinomas, we intended to use miR-200a/b, −141, −205, and −31. Neither of these miRNAs turned out to behave as expected: relative expression changes were uniform yet very subtle for miR-141 whereas the rest of the candidates showed extensive variation in expression restricted to only a minority of tumor samples. Notably, follicular neoplasms consistently displayed reduced relative levels of miR-205 and −31; in contrast, the opposite trend was observed for PTC where these miRNAs were expressed at higher level than in the normal tissue (in 30–40 % of samples).

**Table 2:** Median values of miRNA fold changes in tumors relative to the adjacent non-tumor tissue, as found in different histology-classified tumor subtypes

miRNA	PTC	MTC	FTC	FA	Goiter
21	5.21	3.88	1.35	1.03	1.04
221	16.32	11.42	9.14	1.20	−1.31
222	16.34	11.88	4.30	−1.18	−1.47
205	2.00	−26.13	−12.14	−1.76	−1.47
146b	58.82	−1.62	1.40	−1.35	−1.02
181b	3.70	2.20	1.34	1.22	1.04
200a	1.36	1.71	−1.39	−2.70	−1.92
200b	1.40	1.63	−1.17	−2.42	−1.91
31	6.03	−10.66	−8.39	−3.22	−2.05
187	7.51	2.88	0.07	1.02	−1.38
199b	−2.21	−99.73	−56.15	−39.49	−12.12
141	1.39	−1.07	2.01	1.22	−1.38
144	−4.81	−33.51	−2.97	−3.19	−6.19
192	−1.52	−2.81	0.01	−1.55	−2.38
375	12.93	95.00	−1.66	−4.14	−2.32

### Assessment of the method variation and the choice of the threshold values for microRNA level changes

In order to estimate the variation of the whole procedure of analysis, several pieces of adjacent non-tumor thyroid tissue were lysed, and three equal aliquots of the lyzed material were independently processed according to the RNA extraction protocol. This provided us with an estimate of how much relative levels of each miRNA varied among the technical replicas (Table 3). Note that the values for 95^th^ and 99^th^ percentiles are equal. In different replicas, calculated relative values of miRNA levels turned out to vary up to 5.5-fold, which reflects the maximum level of differences, which could be attributed to purely technical reasons rather than to biological variance.

**Table 3:** Variation of measured levels of different miRNAs in technical replicas from the same tissue sample

miRNA	Median fold change	75^th^ percentile	95^th^ and 99^th^ percentiles
181b	1.28	1.44	1.67
187	1.29	1.43	1.83
205	1.52	1.90	2.39
222	1.51	2.04	2.39
200a	1.67	2.28	2.79
221	1.67	2.44	2.85
146b	1.69	2.28	3.39
199b	1.78	2.79	3.43
21	1.88	2.87	3.56
31	1.64	3.43	5.62

Next, to assess the total measurement error, three different pieces of adjacent non-tumor thyroid tissue from the same patient were independently processed and their values were compared. Table 4 summarizes median fold differences between replicas of a normal thyroid sample from a single donor, as well as 95^th^ and 99^th^ percentiles of the dataset. In our hands, the range of these fold change values typically lies within 1.5-2, however in several instances 10–15 fold difference was observed. Based on these numbers, threshold values of miRNA fold changes were selected (Table 4), so that the values below this threshold would not be considered reliable, as they may be fully attributable to cumulative method variation (including biological variation).

**Table 4:** Fold changes in measured miRNA levels, as assessed for adjacent non-tumor thyroid tissue samples from the same patient

miRNA	Median	95^th^ percentile	99^th^ percentile	Maximum	Threshold fold change value
124	1.71	4.76	5.21	5.21	5.5
221	1.52	4.02	5.16	5.91	6.0
191	1.47	4.66	5.70	5.70	6.0
21	1.46	4.57	5.93	6.31	6.5
31	1.64	4.66	6.06	6.19	6.5
200b	1.60	4.08	5.50	6.02	6.5
181b	1.40	3.18	5.23	7.78	7.0
187	1.48	3.34	4.59	7.57	7.0
192	1.60	4.13	5.82	6.68	7.0
199b	1.57	5.13	7.01	7.84	7.5
222	1.47	3.96	6.23	7.18	7.5
200a	1.62	4.59	7.21	8.40	8.0
205	1.55	4.48	7.24	8.00	8.0
146b	1.62	4.84	8.31	8.91	8.5
141	1.55	5.21	7.73	8.63	8.5
144	1.67	4.68	9.58	9.99	10.0
375	2.03	11.08	15.35	16.80	15.5

### Diagnostic characteristics of relative miRNA level changes

In each neoplasm subtype we estimated diagnostic characteristics of individual miRNA fold changes as stand-alone markers, using histopathology report as a reference method and the selected threshold values as cutoffs. In Table 5, the results for the most informative miRNAs are shown. Sensitivity indicates in what percentage of samples related to some type of neoplasm, according to the histological conclusion, recorded a significant change in the amount of the miRNA. It should be noted that the maximum sensitivity values (80–90 %) are characteristic of carcinomas (leaving MTC out of consideration due to the small amount of samples), in benign neoplasms the change in the level of miRNA is mostly recorded in 20–50 % of the samples.

**Table 5:** Diagnostic characteristics of relative miRNA expression levels as applied to thyroid cancer subtyping

Cancer type	miRNA	Expression level	Specificity (%)	Sensitivity (%)	PPV (%)	NPV (%)
PTC (*n* = 108)	21	Up	99.00	37.96	97.62	59.64
	221	Up	83.00	81.48	83.81	80.58
	222	Up	92.00	75.93	91.11	77.97
	205	Up	98.99	26.85	96.67	55.37
	146b	Up	100.00	88.89	100.00	89.29
	181b	Up	95.00	27.78	85.71	54.91
	31	Up	100.00	48.45	100.00	65.28
	187	Up	90.00	50.00	84.38	62.50
	375	Up	94.90	49.06	91.23	63.27
MTC (*n* = 4)	21	Up	79.90	25.00	2.38	98.19
	221	Up	50.00	75.00	2.86	99.03
	222	Up	57.35	75.00	3.33	99.15
	205	Up	83.25	75.00	8.11	99.41
	31	Down	84.49	75.00	9.38	99.37
	187	Up	69.12	25.00	1.56	97.92
	199b	Down	47.06	100.00	2.70	100.00
	144	Down	74.51	100.00	5.45	100.00
	375	Up	72.64	66.67	3.51	99.32
FTC (*n* = 16)	221	Up	51.04	68.75	10.48	95.15
	222	Up	55.21	25.00	4.44	89.83
	205	Down	86.39	68.75	29.73	97.06
	31	Down	86.36	53.33	25.00	95.60
	187	Up	68.75	25.00	6.25	91.67
	199b	Down	49.74	93.75	13.51	98.96
FA (*n* = 41)	205	Down	83.23	22.50	24.32	81.76
	200a	Down	90.97	37.84	50.00	85.98
	31	Down	86.36	29.73	34.38	83.65
	199b	Down	51.20	73.17	27.03	88.54
	144	Down	73.49	26.83	20.00	80.26
	200b	Down	87.95	34.15	41.18	84.39
Goiter (*n* = 39)	200a	Down	87.10	21.62	28.57	82.32
	31	Down	84.31	21.05	25.00	81.13
	199b	Down	46.43	53.85	18.92	81.25
	144	Down	75.60	35.90	25.45	83.55
	192	Down	95.24	20.00	50.00	83.33
	200b	Down	86.31	28.21	32.35	83.82

*PPV* positive predictive value, *NPV* negative predictive value

### miRNA classifier for molecular subtyping of thyroid neoplasms

As can be seen from the Table 5, the increase in the relative levels of single miRNAs (miR-146b and −31) can serve as a possible criterion for differential typing of PTC. However, the rest of the tested miRNAs failed to show high sensitivity and specificity values. One could expect that neoplasm types could be more reliably discriminated based on the combinatory information on fold changes of several miRNAs. So we proposed classifier constructed as a decision tree that would help to accomplish this goal, based on the combinatorial patterns of miRNA expression changes. In Table 6, this classifier is provided as well as its estimated diagnostic characteristics.

**Table 6:** Classifier for thyroid neoplasm subtyping based on the combinatorial patterns of miRNA expression changes, and its diagnostic characteristics

Histology-based tumor type	Components of the miRNA pattern	Criterion	Specificity (%)	Sensitivity (%)	PPV (%)	NPV (%)
PTC (*n* = 108)	miR-21, −221, −222, −205, −146b, −31, −187, −181b, −375	Levels of miR-146b or −31 are increased, alternatively levels of at least 3 out of the remaining miRNAs are increased/level of miR-31 is not reduced	98.00	92.59	98.04	92.45
MTC (*n* = 4)	miR-21, −221, −222, −187, −375, −205, −31, −199b, −144,	miR-146b level are not increased At least two miRNAs out of miR-21,- 221, −222, −187, −375 are above norm at least 3 miRNas out of miR-205, −31, −199b, −144 display reduced expression	99.51	75.00	75.0	99.51
FTC (*n* = 16)	miR-221, −222, −187, −205, −31, −199b, −144, −375	Expression levels of miR-21, 146b, 205, 375 are not increased Expression levels of miR-221 or −222 or −187 are increased Expression level of miR-199b is reduced Of miR-205, −31, −144, −375, at least one miRNA shows reduced expression	99.48	81.25	92.86	98.45
FA (*n* = 41)	miR-205, −200a, −200b, −31, −199b, −144 -375	Expression levels of miR-21, −221, −222, −200b are not increased Of miR-181b, −187, −375 one species at most shows increased expression Of miR-205, −200а, −200b, −31, −199b, −144, −375 levels of at least two miRNAs is reduced	86.83	65.85	55.10	91.19
Thyroid cancer (without subtyping) (*n* = 124)	miR-21, −221, −222, −205, −146b, −31, −187, −181b, −375	Expression levels of miR-21, −146b, −221 or −31 are increased, alternatively any two species out of miR-222, −205, −181b, −187, −375 show increased expression levels	96.59	90.83	97.32	88.54

Out of 15 initially selected miRNAs, 13 miRNA species turned out to be informative for thyroid cancer subtyping, namely: miR-221, −222, −146b, −181b, −21, −187, −199b, 144, −200a, −200b, −205, −31, −375. As it follows from the data presented in the Table 6, patterns composed from information on expression changes in 9 miRNAs allow highly accurate discrimination between papillary and medullary thyroid carcinomas and the rest of the thyroid neoplasms, even though our data are somewhat preliminary for MTC due to the small sample size. The lowest PPV and sensitivity were reached for FA. However, the original histopatology report proved to be the least reliable just for FA diagnosis (see section “Second opinion on the samples with conflicting subtyping data” and “Discussion”).

### Detection of BRAF(V600E) mutation

All thyroid neoplasm samples were tested for the presence of BRAF V600E mutation, which was reported to be prevalent in papillary and anaplastic thyroid carcinomas, unlike in FTC and benign thyroid nodules [26–28].

**Fig. 1:**
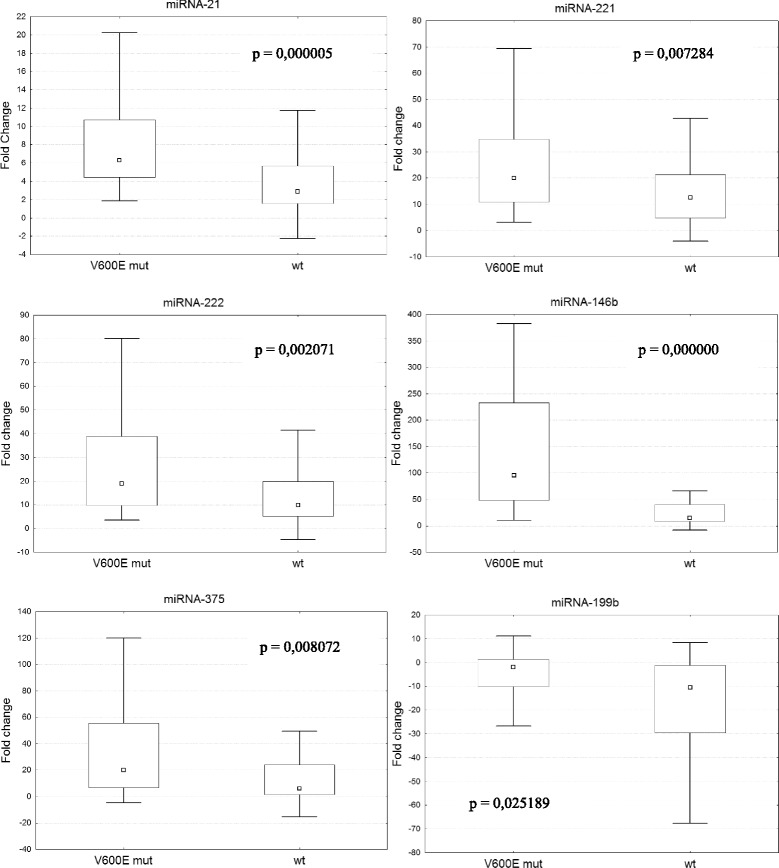
Comparison of fold changes in expression level of selected miRNAs between BRAF(V600E)-positive and negative PTC samples. Square – median, box - interquartile range, whisker – non-outlier range. Statistical significance is evaluated using Mann–Whitney *U* test

BRAF(V600E) mutation was only found in the samples that were classified as PTC by miRNA profiling (61.7 % samples). Out of this samples 61 were classical PTC (67 % of classical PTC), and 5 (31 % of FVPTC) were FVPTC. Importantly, BRAF(V600E)-positive PTC samples were significantly different in the relative expression of six miRNA species from BRAF(V600E)-negative PTCs (Fig. 1). Four of these miRNAs (miR-146b, −21, −221 and −222) were upregulated in all 65 PTC samples from BRAF(V600E) + subgroup, whereas at least one of these miRNAs showed unaltered or even reduced relative expression in some of the samples from BRAF(V600E)- subgroup. In adjacent non-tumor tissues, this mutation was not detected in none of the cases.

### Detection of RET-PTC1 translocation

All samples have been tested for the presence of RET-PTC1 translocation, which is a recognized molecular marker for papillary cancer. This is not the only translocation variant of the RET gene translocation but it appears to be the most common one [29]. RET-PTC1 translocation was only detected in samples of papillary cancer (12 %). Out of this samples 11 were classical PTC (12 % of classical PTCs), and 1 (6 % of FVPTCs) were FVPTC; notably, in tissues adjacent to tumors, in which this translocation was detected, it was also found, albeit in much smaller quantities than in tumor tissues (on average, 70-fold), similar results are mentioned in [30]. In others samples this translocation has not been detected in any adjacent non-tumor tissues. In full accordance with the literature data, in none of the samples we detected the simultaneous presence of BRAF (V600E) mutation and translocation of RET-PTC1.

### Second opinion on the samples with conflicting subtyping data

For some samples, the initial histological report explicitly contradicted the result of the test based on miRNA classifier. Several reasons could have accounted for this issue. On the one hand, our molecular approach could be biased. On the other hand, initial histological analysis is also prone to errors, which include failures to subtype follicular neoplasms and to discriminate between follicular form of papillary carcinoma and truly follicular thyroid neoplasms. In the Russian clinical practice, histological report on the type of thyroid neoplasm is usually made once. Therefore, an error committed by a pathologist can remain undetected throughout the entire treatment of the patient. In order to understand which of these scenarios is the most likely in our study, the samples with conflicting sub-typing data were sent for a blind second opinion to an independent expert pathologist. Data from the first and second histology reports, as well as molecular sub-typing data, are presented in the Table 7.

**Table 7:** Results of the first and second histological reports for 21 thyroid tumor samples whose miRNA profiles conflicted with the first histological analysis

Sample number	Primary diagnosis, histology-based	miRNA profile-based diagnosis	Secondary diagnosis, histology-based	BRAF(V600E) status	RET-PTC1 status
8,15,30,141,184	FA	**PTC** ^a^	**FVPTC**	-	-
28,97	FA	**PTC**	**FVPTC**	**+**	**-**
122,132	Goiter	**PTC**	**FVPTC**	-	-
170	FTC	**PTC**	**PTC**	**+**	**-**
175	Goiter	**PTC**	**PTC**	-	**+**
183	FA	**FTC**	**FTC**	-	-
168	Goiter	**FA**	**FA**	-	-
68	**FA**	FTC	**FA**	-	-
123	**Goiter**	PTC	**Goiter**	**+**	**-**
191	Goiter	FTC	FA	-	-
164	FA	PTC	Goiter	**+**	**-**
106	FA	PTC	Goiter	-	-
5	FA	FTC	Goiter	-	-
179	Goiter	PTC	Goiter/FA	-	-
27	FA	PTC	Goiter/FA	-	-

^a^The criteria used herein fail to discriminate follicular variant of papillary thyroid carcinoma (FVPTC) from the rest of the PTC subtypes

miRNAs shown in boldface type were used in our work

Diagnosis from the secondary pathology report matched the miRNA profiling data for 13 samples (62 %), in one case (Sample No. 175) the second diagnosis and the diagnosis based on miRNA are supported by the detected translocation of RET-PTC1. Only in two instances (9.5 %) the second opinion contradicted miRNA profiling data yet agreed with the initial pathology report. In one of these cases (sample #123, which was twice classified as a goiter), BRAF(V600E) was found, which is typically absent from the benign nodules [31, 32]. In four cases (19 %), the second opinion was in conflict both with the first pathology report and with miRNA typing data; at least for one of these samples (#164), both histology-based conclusions may raise doubts, as BRAF(V600E) mutation was detected and confirmed by 454 sequencing. Finally, in the two remaining samples, the results of second opinion histology analysis were inconclusive.

## Discussion

Data presented in the Table 7 clearly illustrate possible inaccuracies of histological analysis and a contribution of human bias into the final diagnosis and typing of thyroid malignancies. Although medical expert consultation often makes it possible to correct the error of the initial histological report, our practical experience shows that the human factor remains a significant source of diagnostic inefficiency. Limitations of the “classical” morphological analyses, used in both pre- and post-operative subtyping of thyroid tumors are quite well-recognized. Hence many efforts are currently being undertaken to develop molecular diagnostic tools (including those based on miRNA profiling) to sub-type thyroid neoplasms. The list of proposed molecular cancer-specific markers encompasses point mutations in BRAF and RAS; RET/PTC and PAX8/PPARγ rearrangements; sets of miRNAs, for which significant changes in expression have been established for different types of neoplasms; transcripts of protein-coding genes as well as their products, etc. [33–35].

By now, a lot of data has been accumulated about changes in the expression of various miRNA in thyroid tumors. The problem is complicated by the fact that data from different publications are not always consistent with each other and it remains unclear whether miRNA profiling can effectively be used for practical typing of thyroid tumors. This inconsistency may be attributed to several reasons.

- different platforms used to determine miRNA amount in clinical samples can vary in the efficiency of detection of specific individual miRNAs (see, for example, [16, 36–38]). Currently, there is no methodology that would be a “gold standard” in measuring miRNA. The method for extracting the nucleic acid is also essential. It is important to keep in mind, that when measuring the value of relative content of miRNAs in tumor tissue by PCR-based method, a cumulative bias is likely to occur, which is aggravated by the following factors: i) natural (biological) variation of miRNA levels both in tumor tissue and in the adjacent tissues, e.g., age-related changes [39]; ii) sampling errors, i.e., contamination of the normal tissue with tumorous cells, and vice versa, as well as natural heterogeneity of the tissue being analyzed; iii) variance due to handling and storage conditions; iv) variance due to RNA extraction procedure; v) RT and PCR efficiency variance; and vi) not perfect specificity of the test, i.e., the ability to identify only miRNAs of interest, but not the molecules with a similar structure. In our hands, the factors affecting most strongly the cumulative measurement variation were nucleic acid extraction step and heterogeneity of tissue samples. The rest of the factors showed negligible effects on the measurement accuracy (data not shown). We believe that observed magnitude of technical variance (see Tables 3 and 4) is unlikely to be caused by the RNA isolation procedure, so natural variation in miRNA levels, or errors during sample collection may be the contributing factors. These factors should affect the end result regardless of the platform used to measure miRNA. Given that sample collection was beyond our direct control, we had to choose the threshold values so that cumulative contribution from technical errors is minimized.
- Principles of processing raw data obtained in the evaluation of the content of miRNAs in a clinical samples aimed at their integration into the algorithm of typing may be different. This applies to the selection of the normalizer, the principles of calibration in quantitative evaluation, selection of cutoffs for clinically significant values, etc.
- Differences between the results obtained by different groups may result from regional specifics of the samples, or from the study of a too limited number of samples, or of only a few types of tumors. I.e., vast majority of studies employing miRNA profiling for differential diagnostics of thyroid malignancies primarily focus on PTC due to the high prevalence of this lesion subtype and relative ease of diagnostics via morphology means. However, when only the features of papillary carcinoma are analyzed and compared with normal tissue, molecular characteristics common to papillary cancer and other types of neoplasms are overlooked and, therefore, a classifier based on the analysis of samples of PTC may mistake for it other types of thyroid neoplasms.

In our work we did not intend to look for new oncogenic miRNA in thyroid, but used those of them for which the changes in the level have previously been revealed in thyroid neoplasms, selecting a set in such a way as to distinguish between papillary, follicular and medullary thyroid carcinomas and, whenever possible, follicular adenoma.

First, we wanted to determine what results could be obtained on a local sample, because to the best of our knowledge no such study had been carried in Russia before ours, and to compare our findings with literature data. Our data appeared largely in agreement with those obtained by other researchers. Thus, our results fully confirm that the increased level of miR-146b is an exclusive and specific tumor marker for PTC with sufficient sensitivity. On the other hand, in some cases the specificity, which we observed in the changes of the miRNA levels in different types of neoplasms was at variance with the published data. Examples of such differences are presented below:

- miRNA-221, −222 – increase in the number of miRNA in our hands was characteristic of all carcinomas though in varying degrees: PTC – 81.5 % and 76 %, respectively, MTC – about 75 %, FTC – 69 % and 25 %. In [3] an increase in miR-221 in MTCs was not reported.
- miRNA-205 – according to [3] its level should rise for PTC, which is confirmed by our results (the increase was observed in 27 % cases), and in patients with conventional FA, which we cannot confirm. In our study, a reduction in its level has more often been recorded, i.e., MTC – 75 %, FTC – 69 %, FA – 22.5 %, PTC – 9.3 % of cases.
- miRNA-31 – increase in the level of this miRNA was observed for PTC [3, 9] and FA [3], in our sample the increase was observed solely in PTC cases (48 %). On the other hand, its level often decreased in the cases of MTC, FTC (53 %) and of FA (30 %).
- miRNA-199b-5p – this miRNA was chosen to discriminate FA and FTC [12]. Indeed the level of its expression is reduced in 94 % of FTC cases and 73 % FA cases, but also in 100 % MTC cases, 39 % of PTC cases and 54 % of goiters.
- miRNA-375 – Increase in its level tends to be associated with MTC [11], which we can confirm, and also with PTC [10], which is also confirmed by our findings (increased was observed in 49 % cases). Decrease in its level we recorded for FTC in 19 % cases, and in 22 % of FAs. Interestingly, the relative level of miRNA-375 in non-tumor tissue adjacent to the medullar cancer tissue was significantly higher (on average, 68-fold) than in non-tumor tissues adjacent to any other type of neoplasms.
- miRNA-200a, −200b – based on their reported functions [19, 21], we assumed that their level would be reduced to a greater extent for FTC cases. Indeed, more often, their level was reduced (38 and 34 % of FA cases and in 21 and 19 % of FTC cases), although in some samples of PTC (8 and 9 %) and FTC (7 and 6 %) we observed the increase in their content.

These differences in the evaluation of the specificity of changes in the content of certain miRNAs with results of other studies are by no means unexpected. Firstly, we used fairly high threshold values, above which changes in the content of miRNAs we considered reliable (for example, for the miR-146b only an increase in comparison with the control more than 8.5 times was seen by us as reliable). To the best of our knowledge, most of the studies in this field were not considered a variation of the procedure of analysis as a whole, from the dissection. We believed that using high threshold values was to increase the specificity of discrimination of tumor types, perhaps at the cost of some desensitization. Secondly, we used for normalization non-tumor adjacent tissue from the same patient, in which the level of certain miRNAs could in itself have been different from the reference values determined for healthy tissue. We chose this strategy because it is more accurate and practically implementable under the surgical removal of the thyroid tissue for the postoperative typing.

On the basis of the obtained data and taking into account the selected threshold values we have managed to develop a classifier based on the analysis of miRNA and enabling the discrimination of neoplasms with sufficiently high specificity. Indeed, we have succeeded in distinguishing different types of carcinomas from each other and from benign neoplasms with sufficient accuracy. Despite the fact that these criteria may have to be updated as new cases are added, they still make it possible to clarify some controversial situations arising at postoperative typing of neoplasms due to the impact of the human factor and because of the ambiguity of the classification itself, especially regarding follicular tumors.

The problems associated with the discrimination of follicular thyroid neoplasms, especially ETC. and FA are all too known. Our current understanding of the data concerning chain of molecular events accompanying the progress of follicular adenoma into carcinoma is far from complete; neither can we tell which morphological characteristics of this transformation can be definitive for a pathologist. For example, the presence of a mutation in a single isoform of the RAS gene can be indicative of an increased potential for the FA transformation to FTC [40, 41], but in follicular adenomas with detected RAS mutations no clear morphological differences have been identified from those, in which such mutations have not been detected. Thus, we speculate that higher resolution of molecular diagnostic tools, as compared to the histological analysis, may underlie the discrepancy between the results of the two approaches.

The fact that molecular classifiers similar to the one proposed by us demonstrate comparatively inferior diagnostic characteristics with regard to follicular neoplasms can be attributed to the heterogeneity of samples classified histologically such as FA, FTC, or FVPTC. On assessing the diagnostic characteristics of our classifier, the histological report served as a reference method, but it is clear that some samples can initially have been mistyped by histological analysis. Also it should be emphasized that in itself the classification of thyroid tumors is by no means established. Discussions are currently underway suggesting its revision due to the new data relating to molecular genetics of thyroid tumors [17, 42].

It should be noted that in most cases of contradictions between the initial histological report and molecular typing, the initial report was a benign tumor and molecular diagnosis confirmed by the secondary diagnosis was cancer (12 out of 20 cases when our classifier indicated cancer). Only in five cases we found somatic mutations indicative of papillary carcinoma and confirming the correctness of typing via miRNA classifier. Undoubtedly, the list of somatic mutations that occur in thyroid cancer is much wider than these two. It should be emphasized, however, that it is generally assumed that only mutations in the BRAF gene and translocation RET-PTC are specific for PTC (and generated by those anaplastic cancers) while other genetic changes often observed in PTC patients (first of all, mutations of RAS) can also be detected in cases of FA and FTC, and, accordingly, their detection would be unable to demonstrate the error of the pathologist's report.

On analyzing the entire sample, we actually detected BRAF (V600E) mutation and translocation RET-PTC1 translocation only in PTC with a frequency corresponding to the literature data: BRAF (V600E) mutation was found in 61.7 % of PTC samples (it was observed in 30–83 % cases, according to [43], RET-PTC1 translocation – in 12 % cases (occurs in 2.5–87 % cases [44–46]). Thus, these two markers together were found in 2/3 cases of PTC. It should be noted that for all samples with detected mutation, the profile of miRNA expression changes, according to our classifier, clearly points to the PTC. In addition, relative levels of several miRNA species in BRAF(V600E)-positive PTCs are significantly higher than in BRAF(V600E)-negative papillary cancers. This trend has been previously noted for several miRNAs from this list (see [47, 48]), however, other studies fail to support these observations [49, 50]. The inconsistencies found thus far may be attributable to both distinct miRNA detection methods, as well as to the peculiarities of the samples.

The proposed miRNA classifier proved to be the most accurate precisely for PTC. It should be emphasized that PTC represents the majority of malignant neoplasms of the thyroid gland. Most errors of pathology reports are associated with its follicular variant. Thus, in our study, in seven cases of initial pathology reports FVPTC was apparently mistaken for FA. We believe that for papillary cancer, the maximum diagnostic reliability both in the preoperative and postoperative typing using the data of the analysis of molecular markers will be able to exclude most of errors arising when only histological report is taken into account.

The high relevance of using molecular markers for improving the reliability of typing thyroid neoplasms is universally recognized nowadays. Thus, since 2015 ATA recommends to identify genetic markers such as mutations of RAS isoforms, mutation of BRAF V600E, translocation RET-PTC, PAX8-PPARγ in refining uncertain cytology results and to take into account the result of the analysis in the further patient management. In relation to clinical relevance of miRNA deregulation the available information is still insufficient, but it should be emphasized that virtually every case of malignant transformation of thyroid tissue is accompanied by changes in the expression profile of miRNAs. At the same time, even for the best studied type of tumors (PTC) it is not always possible to identify the specific somatic mutations, despite the considerable list of mutations known to date [17]. Moreover, the identification of the entire diversity of these genetic changes, including not only point substitutions, but also large-scale restructuring of the genome requires the use of a complex variety of approaches, while the set of diagnostically significant miRNAs is relatively limited, and may be uniformly analyzed by simple methods, such as those based on PCR.

We believe that molecular typing of thyroid neoplasms based on the use of miRNAs offers practical prospects, because it can rely on a limited set of markers while employing such simple method as RT-PCR, i.e., it does not require special equipment and professionals with significant experience. Undoubtedly, additional studies may be required in order to come to a consensus on what set of RNAs is optimal for characterizing of different types of thyroid tumors. However, even with allowance for the obvious limitations of our work due to a limited sampling and not too wide set of the analyzed markers as well as the absence of multicenter validation of the developed classifier, our results show that typing of thyroid neoplasms using microRNA profiling can be a self-sufficient approach. Specific features of such typing enable it to complement morphological typing even without additional analysis of somatic mutations For example, it was found that, our classifier does not distinguish between classical PTC and its follicular variant, (while the set of characteristic somatic mutations for these variants is significantly different) [17], but their distinction by means of histological analysis presents a certain problem, so that the follicular variant of PTC is often confused with follicular neoplasms, including follicular adenomas.

## Conclusions

Our data, as well as the results from other groups, argue for the practical feasibility of improved typing of thyroid neoplasms via miRNA profiling by means of simple classifiers in addition to the histological analysis. Even if histological analysis includes the parallel identification of somatic oncogenic mutations, the use of microRNAs may imply additional gain in accuracy.

## Additional files

Additional file 1: Sequences of oligonucleotides used in the study. R6G – Rhodamine 6G (Rhodamine 590), BHQ1 – Black Hole Quencher-1, LNA – Locked Nucleic Acid. (XLS 39 kb)

Additional file 2: Patients Characteristics. (XLSX 12 kb)

Additional file 3: Average values of miRNA fold changes in different types of tumors from individual patients relative to the adjacent non-tumor tissue and the result of BRAF V600E testing. Yes – BRAF (V600E) or RET-PTC1 detected, no- BRAF (V600E) or RET-PTC1 not detected. Blank fields denote data missing for different reasons. PTC – papillary thyroid carcinoma, FVPTC – follicular variant of papillary thyroid carcinoma, FA – follicular adenoma, MTC – medullary thyroid carcinoma, FTC – follicular thyroid carcinoma. (XLS 98 kb)

## Abbreviations

FNAB: fine-needle aspiration biopsy
FTC: follicular thyroid carcinoma
FA: follicular adenoma
PTC: papillary thyroid carcinoma
FVPTC: follicular variant of papillary thyroid carcinoma
MTC: medullary thyroid cancer
EMT: epithelial-mesenchymal transition
Cq: quantification cycle
PPV: positive predictive value
NPV: negative predictive value
